# Recognition of Benefactors

**Published:** 1920-03-13

**Authors:** 


### Recognition of Benefactors.

Far too little public notice has been taken of the distinguished deputation which waited upon Mr. Balfour on the subject of Government recognition and reward of efforts in medical and scientific research. A strong case was made out by Sir Watson Cheyne, M.P., chairman of the Medical Parliamentary Committee, and, though Mr. Balfour gave a sympathetic reply and promised to put the matter before the Prime Minister, he, nevertheless, seemed to go out of his way to a needlessly philosophic consideration of the difficulties of defining to whom awards should exactly be made, because of the gradual stages of discovery and research. But why distinguish between the stages if each or any stage is in itself of importance? It is common knowledge that many famous scientists and research workers have conferred great benefits upon their fellows but have had little recognition. An inventor of an industrial process, and the man who patents a method of removing a cork from a bottle or some trivial but ingenious device, as often as not makes a fortune from it. He conceals his knowledge and protects its monetary value for himself by taking out a patent. But Sir Watson Cheyne pointed out what is the truth—namely, that the obligation is laid upon those who make medical research and arrive at valuable discoveries to publish the discoveries for the public benefit; the man in the laboratory thus comes frequently to be left in the lurch.

Some of the instances given of the vital part played by medical science seem commonplace, because they are so obvious. But Sir Watson did well to remind Mr. Balfour, the Government, and the public of some of the things for which medical science is responsible. It made possible the construction of the Panama Canal by showing the way to combat malaria and yellow fever, which had defeated earlier French attempts to construct it. The part played by medical science was of supreme importance in the recent war; it made war possible, or, looked at from a better angle, it lessened the horrors of war by protecting the armies from frightful diseases.

Everyone knows that scientific workers are generally ill-paid in the ordinary course of events, and Mr. Balfour's tale of Cambridge men carrying on research who received less pay than unskilled Corporation workmen could doubtless be paralleled in various directions. It is by no means superfluous in these times to emphasise the point that workers by brain are at least of some consequence to the State, and that workers by hand are not necessarily the lords of creation to whom all must bow. It is an interesting reflection that a body of distinguished scientists should go humbly to the State for a mere £20,000 to finance a scheme of awards for scientific discoveries and be merely assured that the matter should receive attention; whereas, if a trade-union deputation had gone on a determined errand for a far larger sum in extra wages to a class of workers ordinarily competent in ordinary work, some more definite reply would have been demanded, political parties would have entered upon the scenes, the papers would have discussed every detail of it, and it may safely be assumed that the trade union would not have gone away within the time-limit that it would probably have fixed for decision without some sort of satisfaction. Big battalions count for more than big brains now; there is little imagination, or it would be realised that one important medical discovery alone can do more to promote the happiness of mankind than a number of political or social upheavals. The Government found no insuperable difficulty in allocating the pecuniary awards to those who led their country to victory, and it may be hoped that Mr. Balfour will drop the mantle of philosophic doubt on this point, take up the mantle of a man deeply interested in science—as he is—and give his authoritative support to the granting of the modest sum asked for. We must not leave the crowning of our scientists and leaders of thought to the pathetically inadequate Civil List, which many times, by the inclusion of names of great men, has done discredit to a rich nation.

